# Clinical training: a simulation program for phlebotomy

**DOI:** 10.1186/1472-6920-8-7

**Published:** 2008-01-28

**Authors:** Jun-ichi Taniguchi, Kunihiko Matsui, Toshitaka Araki, Kazuhiko Kikawa

**Affiliations:** 1 Department of General Medicine, Kumamoto University Hospital, 1-1-1 Honjo, Kumamoto 860-8556, Japan; 2 Clinical Education Center, Kumamoto University Hospital, 1-1-1 Honjo, Kumamoto 860-8556, Japan

## Abstract

**Background:**

Basic clinical skills training in the Japanese medical education system has traditionally incorporated on-the-job training with patients. Recently, the complementary use of simulation techniques as part of this training has gained popularity. It is not known, however, whether the participants view this new type of education program favorably; nor is the impact of this program known. In this study we developed a new simulation-based training program in phlebotomy for new medical residents and assessed their satisfaction with the program

**Methods:**

The education program comprised two main components: simulator exercise sessions and the actual drawing of blood from other trainees. At the end of the session, we surveyed participant sentiment regarding the program.

**Results:**

There were 43 participants in total. In general, they were highly satisfied with the education program, with all survey questions receiving scores of 3 or more on a scale of 1–5 (mean range: 4.3 – 4.8), with 5 indicating the highest level of satisfaction. Additionally, their participation as a 'patient' for their co-trainees was undertaken willingly and was deemed to be a valuable experience.

**Conclusion:**

We developed and tested an education program using a simulator for blood collection. We demonstrated a high satisfaction level among the participants for this unique educational program and expect that it will improve medical training, patient safety, and quality of care. The development and dissemination of similar educational programs involving simulation for other basic clinical skills will be undertaken in the future.

## Background

Medical education covers a broad spectrum of concepts and skills, involving not only academic and scientific training but also the acquisition of clinical skills related directly to patient care. Traditionally, medical schools in Japan have provided a passive educational experience, focusing more on academics than on hands-on clinical training. For example, although clinical clerkships were introduced to medical schools, allowing students to rotate between each clinical department every few weeks, students were relegated to a bystander role and were not permitted to directly participate in the provision of patient care. Whereas this traditional education system has undoubtedly contributed to the education of medical students, it is not well-suited to assisting today's students and young doctors in the acquisition of necessary clinical skills. In fact, under the traditional system, even the most basic skills involved in patient care could only be learned after medical license certification.

In recent years, medical education in Japan has changed greatly. As part of this change, a national core curriculum has been proposed that advocates a shift in clinical practicum training to participatory clerkships. Moreover, the National Common Achievement Test (CAT), which includes Computer Based Testing (CBT) and Objective Structured Clinical Examination (OSCE), was introduced nationally in 2005 [1]. The CBT assesses the student's scientific knowledge and the OSCE assesses basic clinical skills such as effective communication and physical examination. Medical students must pass both of these examinations to progress to the next step of clinical clerks. Therefore, these examinations not only monitor the quality of medical education but also provide quality assurance in patient care.

Previous reports from European medical schools, however, have cast doubt as to the effectiveness of the clerkship experience alone in providing adequate basic clinical skills training [2]. It has been suggested that even so-called basic techniques, when performed without adequate training, can do physical and psychological damage to the patient [3]. Additionally, informed consent of patients to be treated by clinical clerks would be necessary [4,5]. For example, although phlebotomy, the taking of blood samples from peripheral veins, is a routine procedure it can cause pain and carries a small risk of serious complications. In Japan, students have traditionally been trained in performing phlebotomy on real patients prior to licensing, potentially exposing patients to some risk without consent.

Simulation programs have become increasingly popular as they have the potential to reduce patient risk in clinical training and improve the quality of patient care. One example of a simulation program is the Advanced Cardiac Life Support training course [6], and many other such training programs are being developed in Japan. Although gaining popularity, basic clinical skills training programs remain underused in many Japanese medical education programs. Additionally, whether these programs are effective educational tools and whether participants view their involvement favorably is unknown. Thus, in this study we designed a simulation-based education program of phlebotomy for first-year residents and assessed participant satisfaction with the program as an educational tool. Despite having completed medical school and having passed the national board examination, at this stage residents have had few chances to perform phlebotomy, either simulated or with a patient.

## Methods

### Setting

In April 2006, we conducted a simulation-based phlebotomy education program for first-year residents, in which they received training for drawing blood from the forearm peripheral vein. In addition, the participants had the opportunity to practice on each other at the end of the program. This course was directed by three investigators (JT, KM, and TA), who also acted as trainers for the entire program. The study comprised 43 first-year residents, who were divided into five groups of eight or nine participants each. Each group received identical training sessions, and, prior to the start, the participants were provided with extensive details of the program by the researchers. Each participant submitted a written informed consent both for the education program and for the study. The program and study were approved by the ethics committee of Kumamoto University.

### Simulation-based skills training program

Each program session lasted for 3 hours and was conducted as follows.

**Figure 1:**
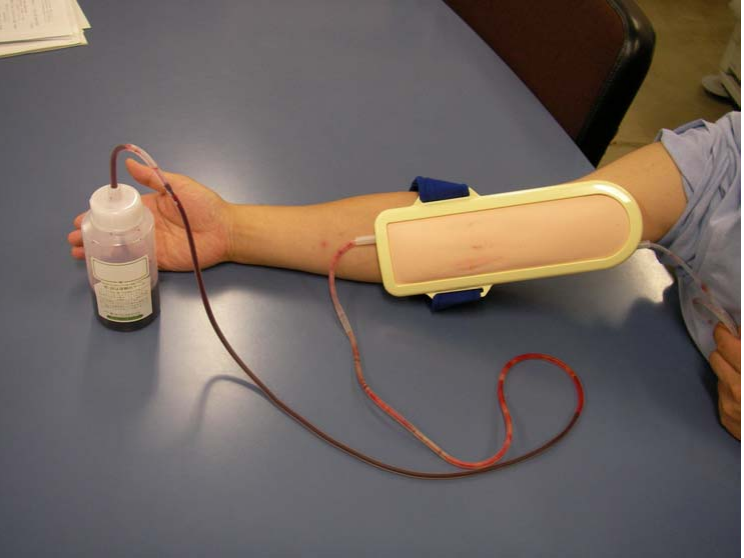
Photograph showing the blood drawing simulator placed on the forearm. Fake blood is drawn using the simulator.

1. *Video orientation session (20 min)*. Each group of participants was shown a video that provided examples of the basic technique used for drawing blood from peripheral veins. Videos were followed by a brief discussion.

2. *Role playing simulation exercise (40 min)*. Each trainee in the patient role was fitted with a phlebotomy simulator (Kantan-kun [Strap-on Vein Puncture Trainer], Kyoto Kagaku, Kyoto, Japan) that was wrapped around their forearm and simulated blood withdrawal was performed (Figure 1). The phlebotomy simulator provided a realistic tissue feel and realistic vein wall resistance that the trainee in the physician role could feel through the needle. During this procedure, the blood-drawing technique and the trainee's communication skills were assessed based on the structured assessment sheet (Table 1). This assessment sheet was prepared by the authors based on their experience and the regulations of this institution.

**Table 1:** Phlebotomy training assessment checklist.

**1. Preparation**
*Preparation of devices and equipment:*
• Preparation of appropriate needle, test tube and other equipment items.
*Confirmation of patient's details:*
• Confirm patient details such as full name, date of birth and address.
**2. Implementation**
*Infection control:*
• Hand-washing.
• Use of hand gloves.
• Placement of sheet under patient's arm.
*Communication with patient:*
• Verbal communication during the procedure.
• Observation of patient's facial expression, e.g., pain.
• Appropriate attitude and provision of explanation, if procedure failed.
*Foresee nerve injury:*
• Ask patient about pain and/or numbness.
• Needle withdrawal.
*Technical skill:*
• Use of strapping band and duration of avascularization (within 2 minutes).
• Angle of needle insertion.
*Arrest bleeding:*
• Confirmation at puncture site and appropriate arrest technique.
**3. After blood is taken**
*Needle-stick accident prevention:*
• Appropriate handling of used devices such as no recap for needle.
*Clear away:*
• Placing each used device in the appropriate location.

Each item was scored as "performed appropriately", "not performed", or "performed but inappropriately" for each point.

3. *Scenario-based simulation (40 min)*. Trainees were paired up and assigned a role playing exercise in which one played the role of the physician and the other the patient in a scenario involving an accident. The trainee in the patient role was provided with information about the "accident" for which they were presenting to the "physician", whereas the "physician" was provided with only minimal information, such as the setting of the accident and the age of the presenting "patient". The "physician" was assessed not only on his or her ability to take blood but also on his or her ability to communicate with the "patient" and obtain pertinent information necessary to make a diagnosis and decide on treatment. All participants played the role of physician and patient at least once. Examples of scenarios that the "physicians" were forced to confront included loss of patient consciousness during blood withdrawal, a patient with hematoma, and a patient with median nerve injury. The cases in each scenario were designed based on the authors' personal experience, expected frequency of such a case, and the degree of injury to the "patient". All role-playing sessions were video recorded [7].

4. *Video Review (30 min)*. All participants watched the video recordings of their role-playing scenarios together and discussed their performances with each other and with the trainers. The trainers tried to elicit the participants' assumptions about the situation and their reasons for acting as they did while in the role of physician [8]. Additionally, the participants playing the role of patient were asked to express their impressions about the behavior and performance of their partner "physician". The participants shared their experiences regarding each scenario.

5. *Peer assessment (15 min)*. The quality of each trainee's blood drawing technique was assessed by their peers based on the same structured assessment sheet used in the previous simulator exercise session (Table 1). This was done prior to the next step of performing actual blood draws on each other. The results were not collected by the researchers but were used to gauge the confidence level of each trainee's peers in their ability to properly draw blood.

6. *Actual blood drawing from other trainees (15 min)*. In the final portion of the educational program, the participants drew blood from each other at most twice, under a trainer's supervision. Whereas training with the simulator was a mandatory part of the medical training program, practicing on other trainees or acting as a subject for actual blood drawing was not, since they would be exposed to some degree of risk of injury. Despite this, all participants completed the entire program.

### Survey, data collection, and analysis

At the end of the session, we anonymously surveyed all participants about their views of the program. Answers to each question were given on a Likert scale of 1 to 5, where a 1 meant fully disagree and a 5 meant fully agree. These results were tallied and displayed using boxplots [9].

## Results

**Figure 2:**
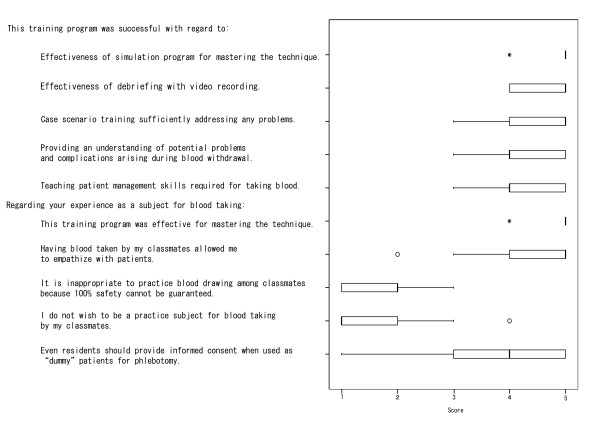
Participant survey questions (left) with the corresponding boxplots of the results (right) (n = 43). 1: Fully disagree, 5: Fully agree. °: Outlier value. *: Extreme outlier value

The survey questions given to each participant in this study along with the corresponding results displayed as boxplots are presented in Figure 2. In general, the participants reported high satisfaction with the program. For example, detailed questions about their impressions produced answers with mean scores ranging from 4.3 to 4.8 on a scale of 5 (5 being the highest level of satisfaction). Moreover, for the question regarding their experience as a real practice subject for their colleagues, the trainees were in agreement that it not only improved their blood drawing technique, but it also simulated the psychological and emotional experiences of their patients.

## Discussion

In this study, we developed and assessed the satisfaction with a new education program on phlebotomy for novice medical residents. This program allowed the residents to experience the blood drawing technique both as a medical professional and as a patient. In general, the participants were highly satisfied with the program.

Our findings are consistent with and extend previous studies from other countries and institutions [4,5,10,11]. At present, we can no longer presume that people will agree to serve as subjects in educational programs [4,5], despite reports such as those by Santen et al. [10,11] that demonstrate a willingness on the part of most patients to allow medical students to perform minor procedures such as suturing, intravenous access, or splinting, even when informed of the student's inexperience. Striking a balance between a minimization of patient risk/concern, while allowing the novice clinician to gain necessary training is an essential part of any educational program to ensure future patient involvement and trainee education. Well-designed training programs will be necessary to accomplish this goal, as will further studies to assess their efficacy.

Educational programs incorporating role playing and simulation are currently recognized as valuable training tools for medical professionals that enable the attainment of practical skills without posing a risk to patients [12-15]. Our report further shows that this type of program is of great value to the trainees themselves. These programs have the capacity to provide learning of a great number of clinical skills. Trainees learn not only how to properly perform a specific clinical technique and how to manage problems that may arise during the procedure, but also to communicate effectively with patients. This study demonstrated that an important way in which trainees may improve their communication skills is through increased empathy with their patients, gained through discussions in which they learned about their "patient's" physical and emotional experiences during the role-playing exercises and by playing the role of patient for their colleagues.

Although it is clear that simulation-based educational programs have great utility in providing valuable training in basic clinical skills, it is obviously not possible to use such programs for the teaching of more invasive procedures, as it would not be possible to guarantee acceptable safety for the participants in such programs. That does not diminish the value of such programs. Rather, the high level of trainee satisfaction and the potential to learn important clinical skills highlights that this type of program should be an integral part of a larger educational system.

Our study had some limitations. First, the study was a cross-sectional survey of a small number of participants at a single institution, limiting the generalization of our results to other institutions and countries. Our results and the responses from participants may have been influenced by their cultural background as well as the Japanese style of education system and medical school programs. Second, the survey questions themselves or the fact that participants were surveyed immediately following the conclusion of the education program may have influenced participants' responses to overestimate the satisfaction level, for example by taking advantage of their feeling of accomplishment. Despite these limitations, however, the present findings indicate that our program is of considerable benefit for inexperienced trainees.

## Conclusion

In conclusion, we have prepared and tested a new training program for phlebotomy, a technique that is one of the most basic and frequently used by medical professionals. The participants played the roles of both medical professional and patient, allowing them to gain the practical skills necessary to properly perform the technique as well as an enhanced empathy for the patient experience. This represents a valuable and unique educational experience for them. This study further shows that this type of program can go a long way towards meeting patient expectations that physicians will be highly skilled in all aspects of the delivery of medical care. Our results suggest that the unique experience of simulation education may contribute to quality improvement of patient care and safety. The design and employment of effective simulation education programs for various other basic clinical skills will further improve training and should be an integral part of the medical school curriculum.

## Competing interests

The author(s) declare that they have no competing interests.

## Authors' contributions

JT and KM conceived of the study. JT, KM, and TA were involved in planning and conducting the education program in the study. JT and KM drafted the manuscript. All authors read and approved the final version of the manuscript.

## Pre-publication history

The pre-publication history for this paper can be accessed here:


